# Activation of endogenous retrovirus triggers microglial immuno-inflammation and contributes to negative emotional behaviors in mice with chronic stress

**DOI:** 10.1186/s12974-023-02724-x

**Published:** 2023-02-15

**Authors:** Han Bao, Jinqi Yan, Jiancheng Huang, Wenjuan Deng, Ce Zhang, Cong Liu, Ailing Huang, Qiao Zhang, Ying Xiong, Qiang Wang, Huanghui Wu, Lichao Hou

**Affiliations:** 1 Department of Anesthesiology, School of Medicine, Xiang’an Hospital of Xiamen University, Xiamen University, No. 2000, East of Xiang’an Rd, Xiamen, 361102 China; 2 Department of Critical Care Medicine, The First Affiliated Hospital of Xi’an Jiaotong University, Xi’an, 710061 China; 3 Department of Anesthesiology, The First Affiliated Hospital of Xi’an Jiaotong University, No. 277, West of Yanta Rd, Xi’an, 710061 China; 4 Translational Research Institute of Brain and Brain-Like Intelligence, School of Medicine, Shanghai Fourth People’s Hospital, Tongji University, Shanghai, 200434 China; 5 Shanghai Key Laboratory of Anesthesiology and Brain Functional Modulation, No.1279, Sanmen Rd, Shanghai, 200434 China

**Keywords:** Endogenous retrovirus, Microglia, Immuno-inflammation, Cyclic GMP-AMP synthase, Stimulator of interferon genes, Chronic stress, Negative emotional behavior

## Abstract

**Background:**

The “missing” link of complex and multifaceted interplay among endogenous retroviruses (ERVs) transcription, chronic immuno-inflammation, and the development of psychiatric disorders is still far from being completely clarified. The present study was aimed to investigate the mechanism of protective role of inhibiting ERVs on reversing microglial immuno-inflammation in basolateral amygdala (BLA) in chronic stress-induced negative emotional behaviors in mice.

**Methods:**

Male C57BL/6 mice were exposed to chronic unpredictable mild stress (CUMS) for 6 w. Negative emotional behaviors were comprehensively investigated to identify the susceptible mice. Microglial morphology, ERVs transcription, intrinsic nucleic acids sensing response, and immuno-inflammation in BLA were assessed.

**Results:**

Mice with chronic stress were presented as obviously depressive- and anxiety-like behaviors, and accompanied with significant microglial morphological activation, murine ERVs genes *MuERV-L*, *MusD*, and *IAP* transcription, cGAS–IFI16–STING pathway activation, NF-κB signaling pathway priming, as well as NLRP3 inflammasome activation in BLA. Antiretroviral therapy, pharmacological inhibition of reverse transcriptases, as well as knocking-down the ERVs transcriptional regulation gene *p53* significantly inhibited microglial ERVs transcription and immuno-inflammation in BLA, as well as improved the chronic stress-induced negative emotional behaviors.

**Conclusions:**

Our results provided an innovative therapeutic approach that targeting ERVs-associated microglial immuno-inflammation may be beneficial to the patients with psychotic disorders.

**Supplementary Information:**

The online version contains supplementary material available at 10.1186/s12974-023-02724-x.

## Introduction

Psychiatric disorders characterized with high recurrence, high mortality, high morbidity, and high diseases burden, affect 970.1 million cases with an increase of 48.1% between 1990 and 2019, and are increasingly recognized as leading causes of global burden [1, 2]. Within a total of 12 psychiatric disorders, anxiety disorders and depressive disorders are most common diseases that are accounted for 301.4 and 279.6 million cases in 2019, respectively [1, 2].

Consistent with the hypothesis that monoamine neurotransmitters dysregulation-associated synaptic plasticity, neuronal death, and neurogenesis abnormality, the focus of mechanism on psychiatric disorders has been dominant on the neuronal aspect. However, a large portion of patients fail to achieve full remission after the first-line antidepressant treatment targeting on regulating monoamine neurotransmitters, indicating that this hypothesis fails to illustrate all aspects of psychiatric disorders. Accumulated evidence from animal and clinical studies suggested the strong association between immuno-inflammation and stress-related psychiatric disorders. The main findings can be summarized as the followings: inflammation and somatic diseases comprising immuno-inflammatory processes increase the risk of psychiatric disorders; pro-inflammatory markers, such as tumor necrosis factor-alpha (TNF-α), interleukin (IL)-1, and IL-6, are generally found at increased levels in the brain, cerebrospinal fluid (CSF), and serum of patients with psychiatric disorders; and pro-inflammatory agents induce psychiatric symptoms, which can be treated with antipsychotics. However, mechanism research in the field of illustrating the original of immuno-inflammation in the central nervous system (CNS) will be described in more detail.

Endogenous retroviruses (ERVs) are relics of ancient retroviral sequences that integrated into the host genome spanning millions of years during species evolution [3]. The detrimental effects of ERVs have been balanced by beneficial activities and become intricate but stable elements between self and foreign DNA, contributing an abundance of genetic novelty and diversity to the host physiological functions. For example, specific ERVs envelope proteins are coopted for pregnancy-related purposes, including maternal–fetal tolerance, placenta-specific cell type formation, trophoblast gene expression network rewiring, and the establishment of imprinting [4], as well as proviral long terminal repeats (LTRs) act as cis-acting regulatory elements that participate in protecting against exogenous infections and regulating various cellular genes transcription [5, 6]. However, the “fossil” ERVs also can be excessively activated and abnormally expressed in some specific pathological and environmental conditions, leading to autoimmune, neurodegenerative, and chronic inflammatory diseases, as well as cancer [7–11]. An interesting line of evidence suggests that ERVs and their products, such as RNA, cytosolic DNA, and proteins, are also the major contributors in shaping and expanding the interferon network, as well as share the bidirectional modulation to the host innate immune system. Moreover, several clinical studies reported that the expression of human ERV-W, Env (envelope), and Gag (capsid/matrix) in the brain, CSF, and serum of patients with psychotic disorders [12–17], suggesting that copies retaining potential to express retroviral proteins or particles could be used to stratify patients with psychotic disorders into subgroups with differing inflammatory and clinical profiles [18, 19].

Given the “missing” link of complex and multifaceted interplay among ERVs transcription and their products expression, chronic immune dysregulation and low-grade of inflammation in the CNS, as well as the development of psychiatric disorders is still far from being completely clarified. Therefore, the current study was aimed to investigate the mechanism of protective role of inhibiting ERVs transcription and their products expression on reversing microglial immuno-inflammation in basolateral amygdala (BLA), the core node within neural network on mediating anxiety and depression, in chronic stress-induced negative emotional behaviors in mice.

## Methods and materials

### Animals

Adult male C57BL/6 mice, aged 6 ~ 8 w, weighted 18 ~ 22 g, were housed in the core animal facility with ambient temperature of 22 ± 1 °C, 30 ~ 70% humidity, 12-h dark/12-h light cycle (light on at 07:00 a.m.), and food and water ad libitum in Xiamen University. Experimental protocol and all procedures were received prior approval from the Institutional Animal Care and Use Committee at Xiamen University (Xiamen, China, XMULAC20190054). Efforts were made to minimize animal suffering and to reduce the number of animals used.

### Chronic unpredictable mild stress (CUMS) mice

CUMS protocol was involved a variety of mild stressors, including 2 h of habitation alone or crowded, 2 h of physical restraint, 2 h of exposure to a foreign object (e.g., a piece of plastic), 5 min of forced swimming at 8 °C, and 10 min of cage shaking during the daytime, as well as food or water deprivation, illumination or flash of light, cage tilt (30°), and habitation in a soiled cage (200 ml of water in 100 g of sawdust bedding) overnight [20]. Mice in CUMS group were daily received 1 of inconsecutive day and night stressors combination that were prior randomly scheduled for a 6-w period. Mice in control group were kept without any stress application in the same housing conditions.

### Adeno-associated virus (AAV) vectors and stereotaxic injection

Mice received inhaled anesthesia with 2% isoflurane (RWD Life Science Co., LTD, Shenzhen, China) and positioned in a stereotaxic instrument (RWD Life Science Co., LTD). For *p53* knocking-down in BLA, pAAV9-U6-mCherry-p53-Mus-169 (titer: 8.38 × 10^13^, 5’ → 3’: GGAGGAGTCACAGTCGGATAT; GenePharma Co., Ltd, Shanghai, China), negative control virus pAAV9-U6-mCherry (titer: 1.04 × 10^13^, 5’ → 3’: TTCTCCGAACGTGTCACGT; GenePharma Co., Ltd) or artificial cerebrospinal fluid (ACSF, containing 150 mM NaCl, 10 mM D-glucose, 10 mM HEPES, 2.5 mM KCl, 1 mM MgCl_2_ (pH 7.35), 312 mOsm) was injected into bilateral BLA (AP, − 1.46 mm; ML, ± 2.80 mm; DV, − 4.75 mm; 0.3 μl in each site) by using a syringe (Hamilton Bonaduz AG, Switzerland) with micropipette (tip diameter ~ 15 µm) attached to a KDS LEGATO 130 micropipette puller (RWD Life Science Co., LTD) at a flow rate of 0.06 µl per min followed by an additional 15 min to allow diffusion of the virus. Mice were housed for 1 w for recovery after AAV injection.

### Antiretroviral therapy and pharmacologically inhibiting reverse transcriptases

Susceptible mice received daily gastric administration of entecavir (0.3 mg/kg in a volume of 0.2 ml; TopScience Co. Ltd., Shanghai, China) for a 2-w antiretroviral therapy. Susceptible mice received daily gastric administration of tenofovir (30 mg/kg in a volume of 0.2 ml; TopScience Co. Ltd.), emtricitabine (200 mg/kg in a volume of 0.2 ml; TopScience Co. Ltd.), lamivudine (100 mg/kg in a volume of 0.2 ml; TopScience Co. Ltd.), or rilpivirine (5 mg/kg in a volume of 0.2 ml; TopScience Co. Ltd.) for a 2-w pharmacologically inhibiting reverse transcriptases.

### Behavioral tests

All mice were habituated to the testing room for 30 min before the start of the test. The test room was dimly illuminated with indirect white lighting, as mice were nocturnal and their innate exploratory behavior was hindered in well-illuminated conditions. Between each test, the arena was cleaned by using 75% ethanol spray to eliminate any residual odors.

#### Open field (OF) test

Mice were placed at the center of a cubic arena (50 cm length, 50 cm width, and 40 cm height). The free locomotion of mice traveled in 10 min was automatically monitored, recorded and analyzed by Noldus EthoVision XT (Noldus Information Technology, Wageningen, Netherlands). The mean velocity was used as a parameter for the locomotion and the percentage of time spent in the center area (center time% = center time/total time × 100%) was a parameter to evaluate animal’s anxiety-like emotion by off-line analysis.

#### Elevated plus maze (EPM) test

Mice were placed at the center of a plus maze facing one of an open arm. The EPM apparatus consisted of two opposing open arms (50 cm length and 10 cm width) and two opposing closed arms with roofless black walls (40 cm height and 60 cm elevated above the floor). The free locomotion of mice traveled in 10 min was automatically monitored, recorded and analyzed by Noldus EthoVision XT (Noldus Information Technology). The percentage of time spent in the open arms [open arms time% = open arms time / (open arms time + closed arms time) × 100%] and the percentage of number of entries in the open arms [open arms entries % = open arms entries / (open arms entries + closed arms entries) × 100%] were the parameters to evaluate animal’s anxiety-like emotion by off-line analysis.

#### Novel object recognition (NOR) test

The NOR test consisted of habituation, training and testing sections, separated by a delay of 30-min length. Briefly, mice were placed at the center of a cubic arena (50 cm length, 50 cm width, and 40 cm height) without any objects for habituation for 5 min before the test. In training section, two identical square woodblocks (2 cm length, 2 cm width, and 2 cm height) were placed in opposite corners of the arena with enough space for mice to move past the woodblocks without interacting with them. In testing section, mice were exposed to one woodblock identical to that presented during training (the familiar object) and another triangle woodblock (2 cm length, 1 cm width, and 2 cm height) not presented during training (the novel object). The investigations of mice sniffing each object (e.g., nose < 2 cm away from object) and rearing on each object in 5 min were automatically monitored, recorded and analyzed by Noldus EthoVision XT (Noldus Information Technology). The discrimination index including the percentage of time spent with the novel object [discrimination index (time) = time spent exploring novel object / time spent exploring both objects] and number of investigations [discrimination index (investigations) = number of investigations for exploring novel object / number of investigations for exploring both objects] was calculated.

#### *Sucrosepreferencetest*

Mice were exposed to sterile water in two identical bottle on training day 1 and to 1% sucrose (Sigma-Aldrich) solution with sterile water on training day 2. The location of two bottles were switched every 12 h to avoid location preference. In testing section, mice were exposed to one bottle of sterile water and the other identical bottle of 1% sucrose solution with 8 h water deprivation, thereafter. The water and sucrose consumption in each bottle for 24 h was recorded and the sucrose preference [sucrose preference% = sucrose consumption / (sucrose consumption + water consumption) × 100%] was calculated.

#### *Forcedswimming(FS)test*

Mice were placed in a transparent cylindrical container (25 cm height with a diameter of 10 cm) filled with warm water (23 ~ 25℃) in 6 min were automatically monitored, recorded and analyzed by Noldus EthoVision XT (Noldus Information Technology). Swimming, struggling, and floating behavior was assessed, the immobility (floating) time in the last 5-min period was recorded, and the percentage of immobility time [immobility time% = immobility time / total time × 100%] was calculated.

#### *Tailsuspension(TS)test*

Mice tails were suspended to an acrylic bar (15 cm length with 30 cm elevated above the floor) in 6 min were automatically monitored, recorded and analyzed by Noldus EthoVision XT (Noldus Information Technology). Escape-related behavior was assessed, the immobility time in the last 5-min suspension period was recorded, and the percentage of immobility time [immobility time% = immobility time / total time × 100%] was calculated.

### Perfusion and immunofluorescence staining

Mice were euthanized and perfused through the ascending aorta with 50 ml of 0.9% normal saline followed by 100 ml of 0.1 M phosphate buffer (PB, pH 7.2 ~ 7.4) containing 4% (w/v) paraformaldehyde. After perfusion, the brain was removed and post-fixed in the same fixative for 2 ~ 4 h and then cryoprotected for 48 h at 4℃ in 0.1 M PB that contained 30% (w/v) sucrose. After being embedded in an inert mounting medium (OCT, Tissue-Tek, Sakura, Torrance, CA, USA), transverse frozen brain sections (50 μm thick) were cut in a cryostat (CM1800; Leica, Heidelberg, Germany) and collected serially. The sections were rinsed in 0.01 M phosphate-buffered saline (PBS, pH 7.2 ~ 7.4) 3 times (10 min each), blocked with 10% fetal bovine serum (FBS) in 0.01 M PBS containing 0.3% (v/v) Triton X-100 for 30 min at room temperature (RT), followed by immunofluorescent labeling.

The brain sections were incubated 4 h at RT and then overnight at 4 ℃ with one or combinations of the following primary antibodies: rabbit anti-Iba-1 antibody (1:200; Abcam) and rat anti-CD68 antibody (1:200; Santa Cruz Biotechnology, Santa Cruz, CA, USA) in the antibody dilution medium containing 0.01 M PBS (pH 7.4) with 5% (v/v) normal donkey serum (PBS-NDS), 0.3% (v/v) Triton X-100, 0.05% (w/v) NaN_3_ and 0.25% (w/v) λ-carrageenan. The brain sections were washed 3 times with 0.01 M PBS (10 min each) and then incubated for 2 h at RT with one or combinations of the following secondary antibody: Alexa 488 donkey anti-rabbit and Alexa 594 donkey anti-rat IgG (1:1000; Abcam) diluted in the antibody dilution medium. The specificity of the staining was tested on the sections from another dish by omitting the specific primary antibody. Finally, the sections were mounted with mounting medium containing DAPI (Abcam) after washing 3 times with 0.01 M PBS (10 min each) as well as obtained and three-dimensional reconstructed by using a confocal laser scan microscope (Zeiss LSM 880 Airyscan; Carl Zeiss Microscopy GmbH, Promenade, Jena, Germany; 1 μm thick optical section).

### Flow cytometry and fluorescence activated cell sorting (FACS)

Microglia harvest and flow cytometry staining was performed according to previous publication [21]. Mice were euthanized and the brains were quickly removed in filtered cold FACS buffer containing 0.01 M PBS with 0.5% FBS after removal of cerebellum and olfactory bulbs on ice, and then meninges, blood vessels and choroid plexus were carefully separated. The brain slices (600 μm thick) containing amygdala were cut on a vibratome (Leica Microsystems), and then bilateral BLA tissues were punched out by a pipette tip with an inner diameter of 1.5 mm and finely minced prior to digestion. Chopped BLA tissues were placed in glass homogenizer with a total volume of 2 ml FACS buffer containing DNase I (50 U; Sigma-Aldrich), papain (50 U; Sigma-Aldrich) and dispase II (3 U; Sigma-Aldrich), and then processed with a mechanic dissociator at 6 rpm for 30 min at RT. Prior to use, papain was activated for 30 min at 37 ℃ and 5% CO_2_. To stop all digestions, samples were diluted with cold FACS buffer and placed on ice. Solutions were then further homogenated for 10 times and filtered through a 70 μm cell strainer (BD Biosciences, NJ, USA). The resulting single cell suspension was centrifuged at 300 × *g* for 10 min at RT.

Percoll (GE Healthcare, USA) gradients were used to remove myelin and enrich the homogenate for viable microglial cells. A stock solution of isotonic Percoll was prepared (9:1 Percoll in 0.1 M PBS). Cell pellets were resuspended in 5 ml of 30% (v/v) SIP diluted with DMEM-F12 (Gibco, ThermoFisher Scientific, Waltham, MA, USA). Cell suspensions were centrifuged without brake for at 300 × *g* 30 min at RT. After centrifugation, cells were collected and washed with cold FACS buffer, centrifuged for 10 min at 300 × *g*, and resuspended and incubated in 1 ml of 1 × red blood cell lysis buffer (Solarbio Life Science) at RT for 5 min. Solutions were centrifuged at 300 × *g* for 10 min and cell pellets were finally resuspended in 100 μl of FACS buffer on ice before flow cytometry staining.

Flow cytometry staining was performed at 4 ℃. To block surface antigens in microglial cells or macrophages, 1 μl of FcR blocking reagent for mouse (1:100; Biolegend, USA) was added to the FACS buffer containing the cell suspension and incubated for 10 min. Subsequently, a mix containing fluorochrome-conjugated antibodies recognizing mouse CD11b-FITC, CD45-PE, I-A/I-E-BV421 (1:200; BD Biosciences). Samples were washed with 500 μl of FACS buffer and centrifuged for 5 min at 300 × *g*. Finally, cell pellets were resuspended in 250 μl of FACS buffer and samples were immediately used for flow cytometry by Cytoflex LX (Beckman Coulter, Inc., CA, USA) and/or FACS by MoFlo Astrios EQS Cell Sorter (Beckman Coulter, Inc.).

### RNA extraction and quantitative reverse transcription PCR (qRT-PCR)

All reagents, buffers, tips, and containers used for qRT-PCR were RNA-free. Total RNA was extracted and purified with TRIzol Reagents (Invitrogen, ThermoFisher Scientific) according to the manufacture’s instructions. The cDNA synthesis using iScript™ Reverse Transcription Supermix (Bio-Rad Laboratories Ltd., USA) and qRT-PCR was performed with CFX96 Touch™ Real-Time PCR Detection System and software (Bio-Rad Laboratories Ltd.). After incubation at 95 ℃ for 5 min, samples were subjected to 40 cycles of 95 ℃ for 10 s and 60 ℃ for 30 s. Relative transcripts expression was determined by calculating the fold change difference in the gene of interest relative to reference gene *GAPDH* and analyzed according to 2-∆∆CT method. Related primers were listed as the followings: *MuERV-L* (Forward primer: TTTCTCAAGGCCCACCAATAGT; Reverse primer: GACACCTTTTTTAACTATGCGAGCT), *MusD* (Forward primer: GATTGGTGGAAGTTTAGCTAGCAT; Reverse primer: TAGCATTCTCATAAGCCAATTGCAT), *IAP* *Pol* (Forward primer: CTTGCCCTTAAAGGTCTAAAAGCA; Reverse primer: GCGGTATAAGGTACAATTAAAAGATATGG), *GAPDH* (Forward primer: CAAAATGGTGAAGGTCGGTGTG; Reverse primer: TGATGTTAGTGGGGTCTCGCTC) [22].

### Western blotting

Microglia in bilateral BLA was harvested by FACS and homogenized with a hand-hold pestle in RIPA lysis buffer (40 μl/1 × 10^5^ cells; Solarbio Life Science) containing 2% sodium dodecyl sulfate (SDS; Solarbio Life Science) and a proteinase and phosphatase inhibitor cocktail (Solarbio Life Science). Protein concentration of each sample was quantified using the BCA assay (Pierce Chemical, Rockford IL), and then the electrophoresis samples were heated at 100 ℃ for 10 min and loaded onto 10% SDS–polyacrylamide gels with standard Laemmli solutions (Bio-Rad Laboratories, CA, USA). After the proteins were electroblotted onto a polyvinylidene difluoride membrane (PVDF, Immobilon-P, Millipore, Billerica, MA, USA), the membranes were placed in a blocking solution containing Tris-buffered saline with 0.02% Tween (TBS-T; Sigma-Aldrich) and 5% non-fat dry milk (Sigma-Aldrich), and incubated 60 min under gentle agitation at RT, then 4℃ for overnight with rabbit anti-p53 antibody (1:1000; Cell Signaling Technology, Inc., MA, USA), rabbit anti-p-p53 antibody (1:1000; Cell Signaling Technology, Inc.), rabbit anti-IFI16 antibody (1:1000; Beyotime Biotechnology, Shanghai, China), rabbit anti-STING antibody (1:1000; Beyotime Biotechnology), rabbit anti-NF-κB p65 antibody (1:1000; Cell Signaling Technology, Inc.), rabbit anti-p-NF-κB p65 antibody (1:1000; Cell Signaling Technology, Inc.), rabbit anti- NLRP3 antibody (1:200; Cell Signaling Technology, Inc.), rabbit anti- caspase-1 antibody (1:1000; Cell Signaling Technology, Inc.), rabbit anti-IL-1β antibody (1:1000; Cell Signaling Technology, Inc.), and mouse anti-β-actin antibody (1:5000; Sigma-Aldrich), respectively. Bound primary antibodies were detected by incubating with anti-mouse or anti-rabbit horseradish peroxidase (HRP)-conjugated secondary antibody (1:10,000; Amersham Pharmacia Biotech Inc., Piscataway, NJ, USA) for 2 h under gentle agitation at RT. Between each step, the immunoblots were rinsed with TBS-T for 3 times (10 min each). Protein blots’ densities were detected by using enhanced chemiluminescence (ECL; Solarbio Life Science) and analyzed in the Bio-Rad ChemiDoc Imaging System (Bio-Rad Laboratories Ltd.). Immunoreactive bands were quantified and normalized to β-actin.

### Statistical analysis

Data were expressed as mean ± standard error mean (SEM). One-way or repeated measure analysis of variance (ANOVA) followed by Tukey’s post hoc test was used for multiple comparison. The area under the time-course curves (AUCs) values during the analysis time was used to measure the summed effects of body weight. Sholl analysis was applied to analyze microglial morphology by counting the number and length of branch tips with ImageJ (http://imagej.net). All these data were analyzed by using GraphPad Prism version 8.3.0 for Windows (Graph Pad Software, San Diego California USA, www.graphpad.com). *P* < 0.05 was considered as statistical significance.

## Results

### Antiretroviral therapy entecavir reversed chronic stress-induced negative emotional behaviors

**Fig. 1:**
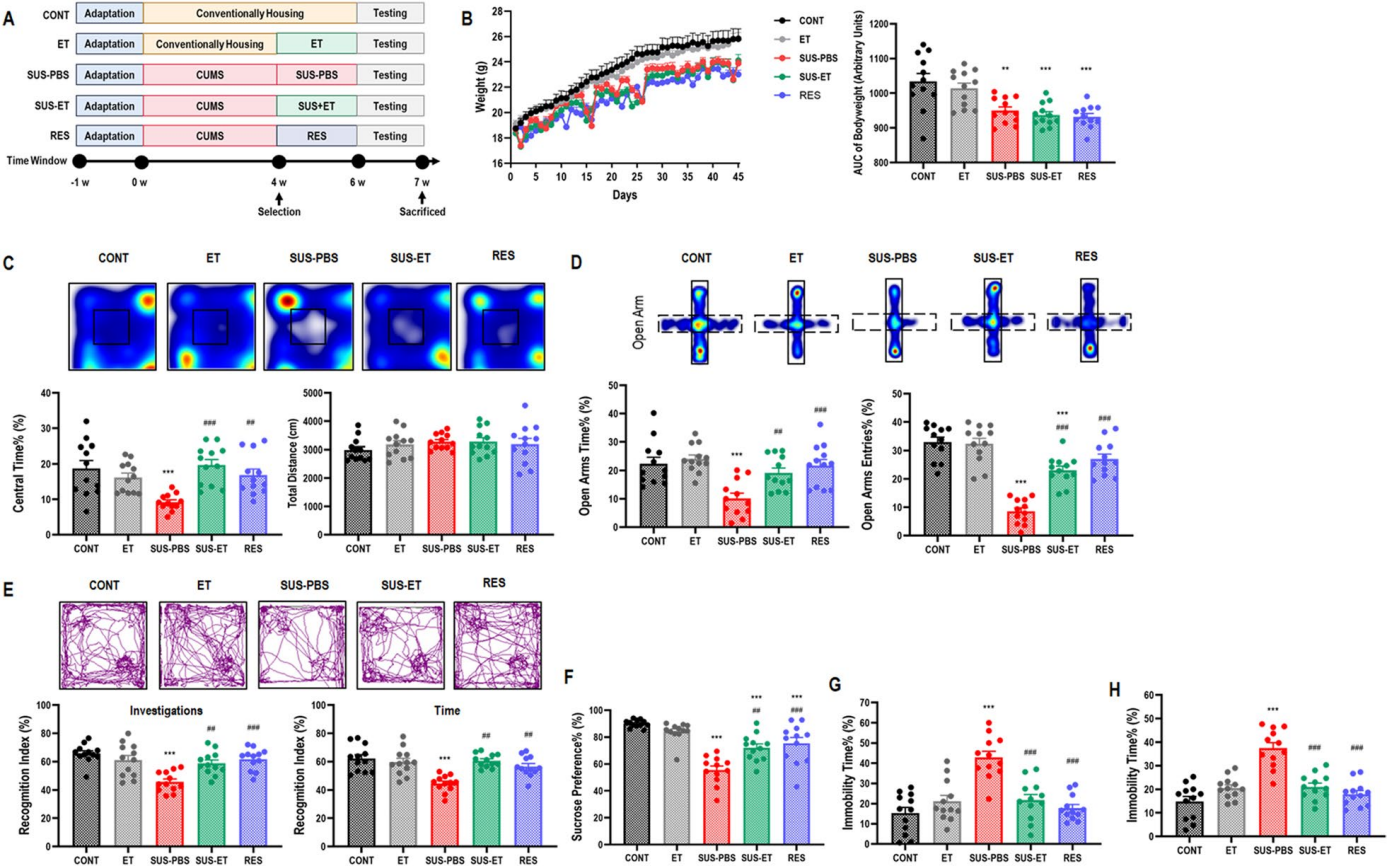
Antiretroviral therapy entecavir reversed chronic stress-induced negative emotional behaviors. **A** Schematic representation. **B** Diagram showing the daily bodyweight, as well as statistical analysis of bodyweight during a 6-w period for each group. One-way ANOVA with Tukey's post hoc test, **P < 0.01, compared with CONT; *n* = 12. **C** Heat maps and statistical analysis of central time% and total distance in OF test for each group. One-way ANOVA with Tukey's post hoc test, ***P < 0.001, compared with CONT; ##*P* < 0.01, ###*P* < 0.001, compared with SUS-PBS; *n* = 12. **D** Heat maps and statistical analysis of open arms time% and open arms entries% in EPM test for each group. One-way ANOVA with Tukey's post hoc test, ***P < 0.001, compared with CONT; ##*P* < 0.01, ###*P* < 0.001, compared with SUS-PBS; *n* = 12. **E** Track maps and statistical analysis of recognition index of investigations and time in NOR test for each group. One-way ANOVA with Tukey's post hoc test, ***P < 0.001, compared with CONT; ##*P* < 0.01, ###*P* < 0.001, compared with SUS-PBS; *n* = 12. **F** Statistical analysis of sucrose preference% in SP test for each group. One-way ANOVA with Tukey's post hoc test, ***P < 0.001, compared with CONT; ##*P* < 0.01, ###*P* < 0.001, compared with SUS-PBS; *n* = 12. **G** Statistical analysis of immobility time% in FS test for each group. One-way ANOVA with Tukey's post hoc test, ***P < 0.001, compared with CONT; ###*P* < 0.001, compared with SUS-PBS; *n* = 12. **H** Statistical analysis of immobility time% in TS test for each group. One-way ANOVA with Tukey's post hoc test, ***P < 0.001, compared with CONT; ###*P* < 0.001, compared with SUS-PBS; *n* = 12

Patients with chronic stress are usually accompanied with negative emotion, such as anxiety and depression. When mice were experienced a 6-w of CUMS (Fig. 1A), all were presented a slower daily bodyweight gain, no matter they were susceptible (approximately 63.8%) or resistant to chronic stress (Fig. 1B). Compared with control and resistant mice, susceptible mice presented significant negative emotional behaviors indicated by decreased center time% in OF test (Fig. 1C), decreased open arms time% and open arms entries% in EPM test (Fig. 1D), decreased recognition index on investigations and time in NOR test (Fig. 1E), decreased sucrose preference% in SP test (Fig. 1F), as well as increased immobility time% in both FS and TS test (Fig. 1G, H).

Interestingly, susceptible mice receiving a 2-w antiretroviral therapy Entecavir strikingly reversed the negative emotional behaviors in all of the parameters described above (Fig. 1C–H). However, no obvious positive effect on daily bodyweight gain, compared with control and susceptible mice (Fig. 1B). Besides, the blood routine test was applied to examine the safety of antiretroviral therapy (Additional file 1: Table S1).

### Antiretroviral therapy entecavir reversed chronic stress-induced microglial morphological activation, ERVs transcription, intrinsic nucleic acids sensing response, and immuno-inflammation in BLA

**Fig. 2:**
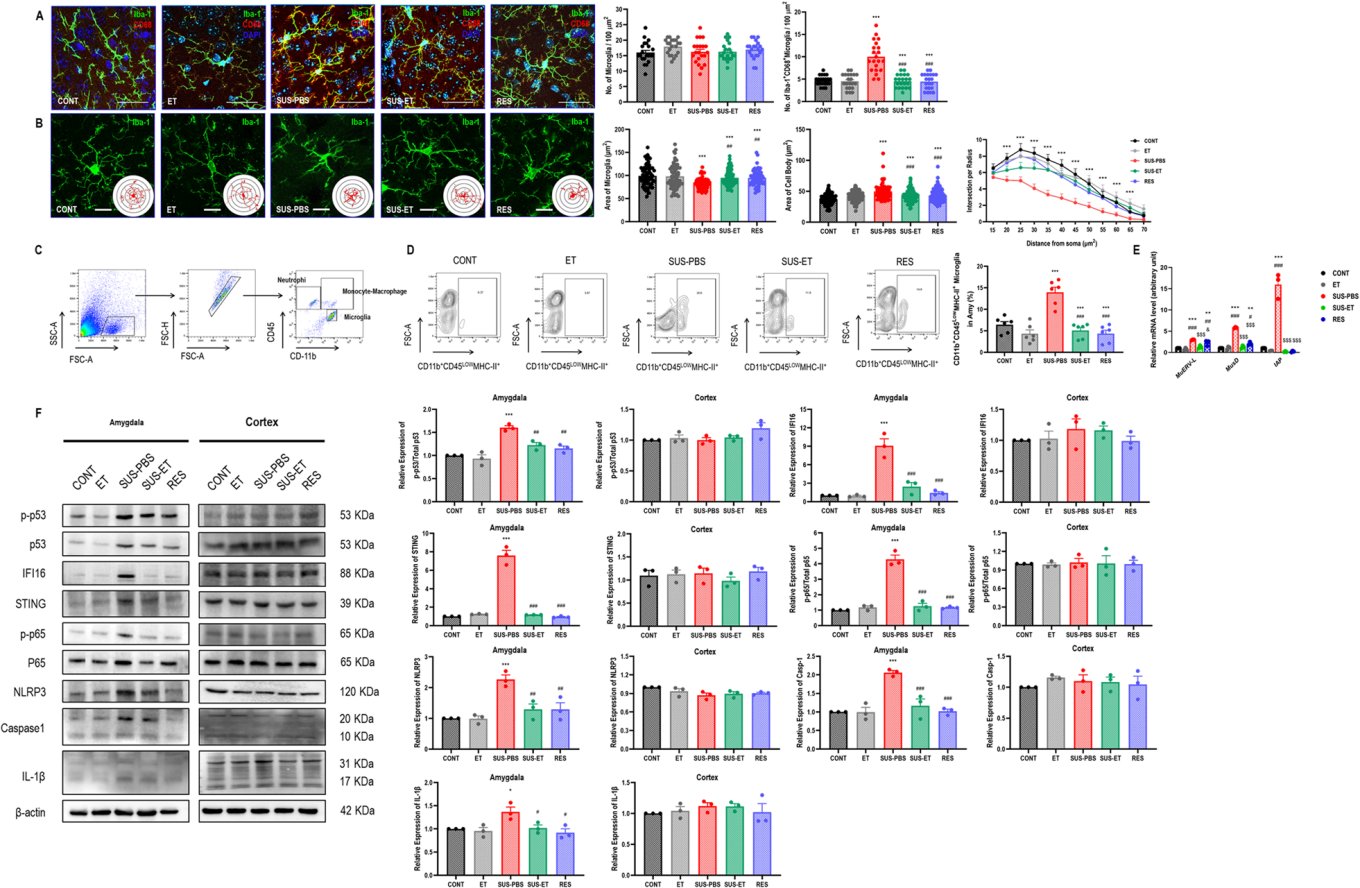
Antiretroviral therapy Entecavir reversed chronic stress-induced microglial morphological activation, ERVs transcription, intrinsic nucleic acids sensing response, and inflammation in BLA. **A** Representative immunofluorescent images showing Iba1^+^CD68^+^ microglia in BLA, as well as statistical analysis of the number of Iba1^+^ microglia and Iba1^+^CD68^+^ microglia in BLA. One-way ANOVA with Tukey's post hoc test, ***P < 0.01, compared with CONT; ###*P* < 0.001, compared with SUS-PBS; scale bar = 40 × ; a total of 21 replicates from *n* = 3. **B** Representative immunofluorescent and their 3D reconstructive Sholl images showing Iba1^+^ microglia in BLA, statistical analysis of microglial area and cell body area of Iba1^+^ microglia in BLA, as well as Sholl analysis of Iba1^+^ microglia in BLA. One-way ANOVA for areas analysis and repeated measure ANOVA for Sholl analysis with Tukey's post hoc test, ***P < 0.01, compared with CONT; ##*P* < 0.01, ###*P* < 0.001, compared with SUS-PBS; scale bar = 60 × ; a total of 90 replicates from *n* = 3. **C** Representative flow cytometry strategy for gating CD11b^+^CD45^Low^ microglia in BLA. **D** Representative flow cytometry images for showing CD11b^+^CD45^Low^MHC-II^+^ microglia in BLA, as well as statistical analysis of percentage of CD11b^+^CD45^Low^MHC-II^+^ microglia in BLA. One-way ANOVA with Tukey's post hoc test, ***P < 0.001, compared with CONT; ###*P* < 0.001, compared with SUS-PBS; *n* = 6. **E** Statistical analysis of murine orthologous microglial ERVs genes expression in BLA. One-way ANOVA with Tukey's post hoc test, $$$*P* < 0.01, compared with ET; #*P* < 0.05, ##*P* < 0.01, ###*P* < 0.001, compared with SUS-PBS; *n* = 3. **F** Representative western blot images for showing microglial p-p53, p53, IFI16, STING, p-p65, p65, NLRP3, caspase-1, IL-1β, and β-actin in BLA and cortex, as well as their statistical analysis of relative proteins expression. One-way ANOVA with Tukey's post hoc test, *P < 0.05, ***P < 0.001, compared with CONT; #*P* < 0.05, ##*P* < 0.01, ###*P* < 0.001, compared with SUS-PBS; *n* = 3

Given that microglia are the key resident immune cells mediating immuno-inflammatory process in central nervous system, therefore, we investigated the microglial morphological activation and biological immuno-inflammation. The morphological results showed that microglia presented as pro-inflammatory activation indicated by the increased percentage of Iba1^+^CD68^+^ microglia with a shrunken area of cells, enlarged cell bodies, and less intersection of branches in BLA in susceptible mice, compared with control and resistant mice (Fig. 2A, B). For more specifically identifying the role of microglia, we further investigating biological immuno-inflammation in CD11b^+^CD45^Low^ microglia with FACS (Fig. 2C). The results showed that the increased percentage of CD11b^+^CD45^Low^MHC-II^+^ microglia, compared with control and resistant mice (Fig. 2D) together with the upregulation of murine microglial ERVs genes *MuERV-L*, *MusD*, and *IAP* (Fig. 2E). Moreover, we harvested microglia in amygdala and cortex of susceptible mice for western blot to investigate the immuno-inflammatory signaling pathways. The results showed that microglial intrinsic nucleic acids sensing response cyclic GMP-AMP synthase (cGAS)–interferon inducible 16 (IFI16)–stimulator of interferon genes (STING) activation, nuclear factor-kappa B (NF-κB) signaling pathway priming, as well as NOD-, LRR- and pyrin domain-containing 3 (NLRP3) inflammasome activation in BLA, instead of those in cortex, in susceptible mice, compared with control and resistant mice (Fig. 2F). Interestingly, the repression of repetitive elements is an important facet of tumor-suppressor gene *p53*'s function as a guardian of the genome, however, paradoxically, pharmacologic activation of *p53* significantly induced the transcription of ERVs via increased occupancy on ERVs promoters and inhibition of two major ERVs repressors, histone demethylase LSD1 and DNA methyltransferase DNMT1 [23]. We also found the significantly upregulated expression of p-p53 in susceptible mice, compared with control and resistant mice (Fig. 2F).

Importantly, susceptible mice receiving a 2-w antiretroviral therapy Entecavir strikingly revered microglial morphological activation (Fig. 2A, B), decreased the percentage of CD11b^+^CD45^Low^MHC-II^+^ microglia (Fig. 2D), downregulation of *MuERV-L*, *MusD*, and *IAP* (Fig. 2E), as well as inhibited microglial cGAS–IFI16–STING activation, NF-κB signaling pathway priming, as well as NLRP3 inflammasome activation in BLA, compared with susceptible mice (Fig. 2F). However, no additional impact on microglial p-p53 expression in BLA of entecavir-treated mice, compared with susceptible mice (Fig. 2F).

### Pharmacologically inhibiting reverse transcriptases reversed chronic stress-induced negative emotional behaviors

**Fig. 3:**
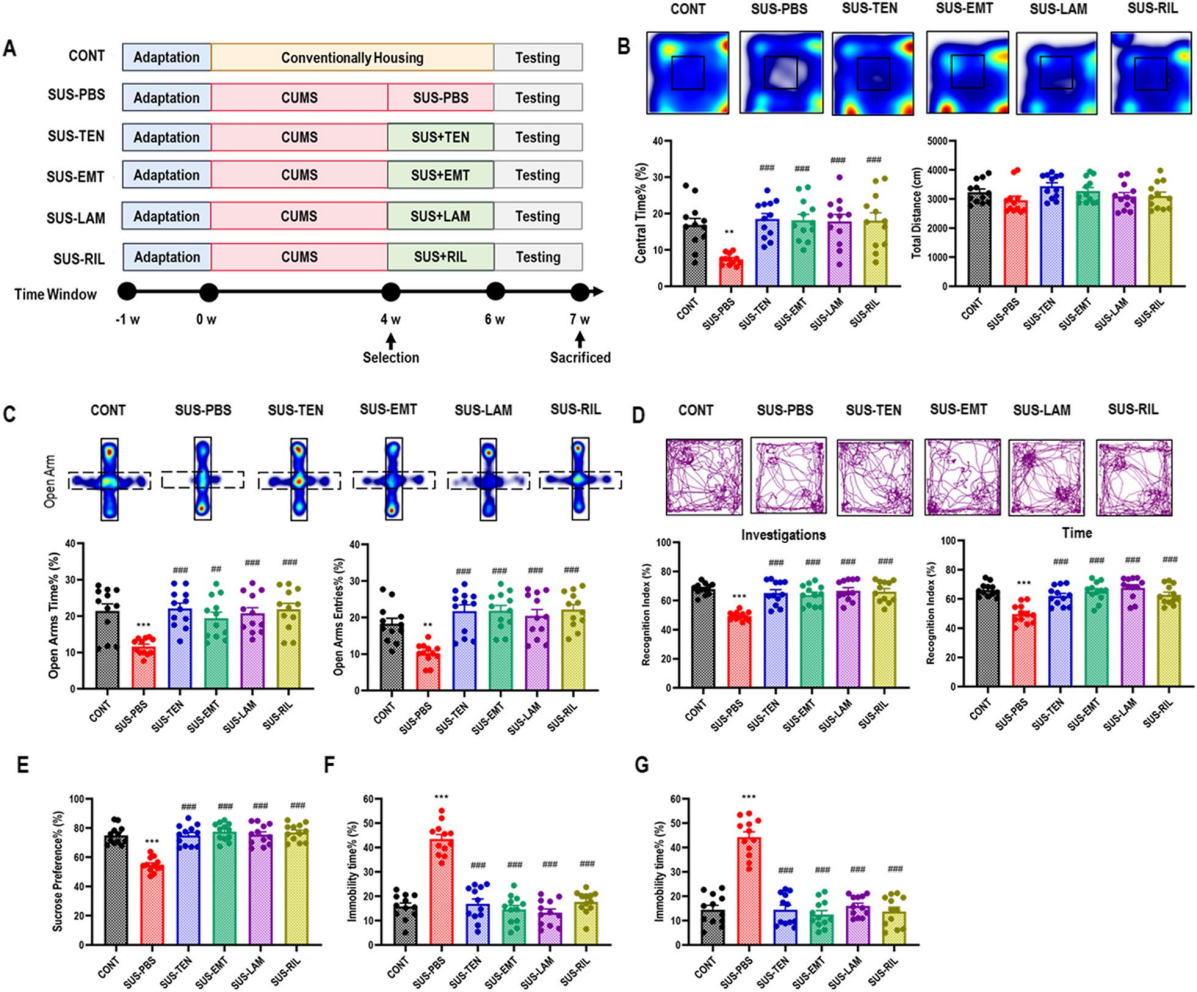
Pharmacologically inhibiting reverse transcriptases reversed chronic stress-induced negative emotional behaviors. **A** Schematic representation. **B** Heat maps and statistical analysis of central time% and total distance in OF test for each group. One-way ANOVA with Tukey’s post hoc test, **P < 0.01, compared with CONT; ###*P* < 0.001, compared with SUS-PBS; *n* = 12. **C** Heat maps and statistical analysis of open arms time% and open arms entries% in EPM test for each group. One-way ANOVA with Tukey’s post hoc test, **P < 0.01, ***P < 0.001, compared with CONT; ##*P* < 0.01, ###*P* < 0.001, compared with SUS-PBS; *n* = 12. **D** Track maps and statistical analysis of recognition index of investigations and time in NOR test for each group. One-way ANOVA with Tukey’s post hoc test, ***P < 0.001, compared with CONT; ###*P* < 0.001, compared with SUS-PBS; *n* = 12. **E** Statistical analysis of sucrose preference% in SP test for each group. One-way ANOVA with Tukey’s post hoc test, ***P < 0.001, compared with CONT; ###*P* < 0.001, compared with SUS-PBS; *n* = 12. **F** Statistical analysis of immobility time% in FS test for each group. One-way ANOVA with Tukey’s post hoc test, ***P < 0.001, compared with CONT; ###*P* < 0.001, compared with SUS-PBS; *n* = 12. **G** Statistical analysis of immobility time% in TS test for each group. One-way ANOVA with Tukey’s post hoc test, ***P < 0.001, compared with CONT; ###*P* < 0.001, compared with SUS-PBS; *n* = 12

The most recent of ancient infections, attributed to ERVs, such as the human ERV-K, have led to preserved provirus sequences that are partially intact and can code for all the viral proteins with transcriptional activity [24, 25]. Recombination or trans-complementation between ERVs’ products, including viral RNA, proteins, and virus-like particles, could lead to replication-competent viruses, suggesting that the viral genome replicated through reverse transcription under some certain circumstances [26–30]. Considering these biological process is regulated by a series of reverse transcriptase, we further identified the potential role of pharmacologically inhibiting the key enzymes of reverse transcription of ERVs’ products on negative emotional behaviors. The behavioral results showed that susceptible mice receiving a 2-w adenine analog acyclic nucleoside reverse transcriptase inhibitor (NRTI) tenofovir, cytosine analog acyclic NRTI emtricitabine, non-acyclic NRTI (Lamivudine), as well as non-NRTI (NNRTI) rilpivirine (Fig. 3A) significantly reversed the negative emotional behaviors indicated by increased center time% in OF test (Fig. 3B), increased open arms time% and open arms entries% in EPM test (Fig. 3C), increased recognition index on investigations and time in NOR test (Fig. 3D), increased sucrose preference% in SP test (Fig. 3E), as well as decreased immobility time% in both FS and TS test (Fig. 3F, G).

### Pharmacologically inhibiting reverse transcriptases reversed chronic stress-induced microglial morphological activation, ERVs transcription, intrinsic nucleic acids sensing response, and immuno-inflammation in BLA

**Fig. 4:**
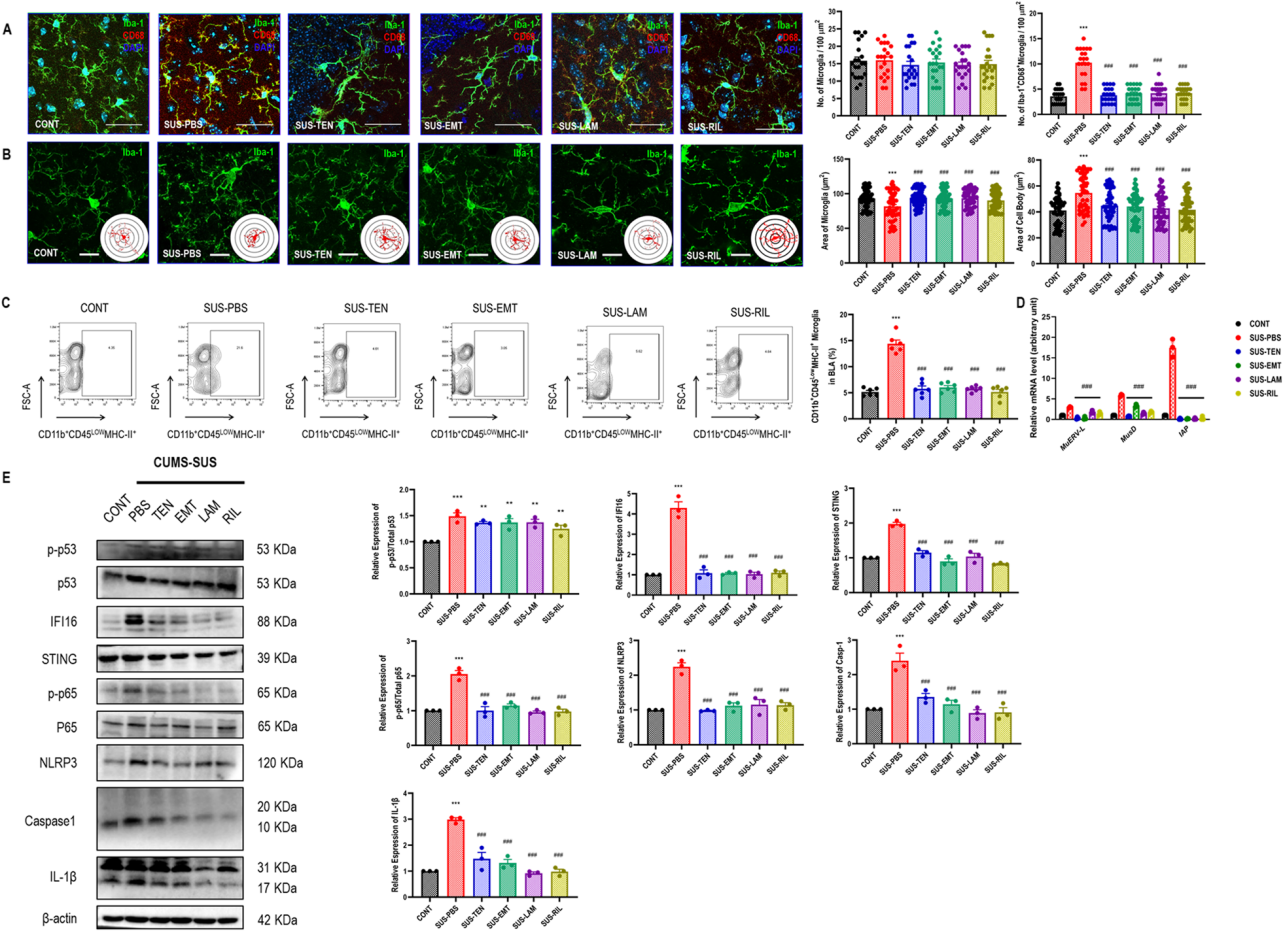
Pharmacologically inhibiting reverse transcriptases reversed chronic stress-induced microglial morphological activation, ERVs transcription, intrinsic nucleic acids sensing response, and inflammation in BLA. **A** Representative immunofluorescent images showing Iba1^+^CD68^+^ microglia in BLA, as well as statistical analysis of the number of Iba1^+^ microglia and Iba1^+^CD68^+^ microglia in BLA. One-way ANOVA with Tukey’s post hoc test, ***P < 0.01, compared with CONT; ###*P* < 0.001, compared with SUS-PBS; scale bar = 40 ×; a total of 21 replicates from *n* = 3. **B** Representative immunofluorescent and their 3D reconstructive Sholl images showing Iba1^+^ microglia in BLA, as well as statistical analysis of microglial area and cell body area of Iba1^+^ microglia in BLA. One-way ANOVA with Tukey’s post hoc test, ***P < 0.01, compared with CONT; ###*P* < 0.001, compared with SUS-PBS; scale bar = 60 ×; a total of 90 replicates from *n* = 3. **C** Representative flow cytometry images for showing CD11b^+^CD45^Low^MHC-II^+^ microglia in BLA, as well as statistical analysis of percentage of CD11b^+^CD45^Low^MHC-II^+^ microglia in BLA. One-way ANOVA with Tukey’s post hoc test, ***P < 0.001, compared with CONT; ###*P* < 0.001, compared with SUS-PBS; *n* = 6. **D** Statistical analysis of murine microglial ERVs genes expression in BLA. One-way ANOVA with Tukey’s post hoc test, ###*P* < 0.01, compared with SUS-PBS; *n* = 3. **E** Representative western blot images for showing microglial p-p53, p53, IFI16, STING, p-p65, p65, NLRP3, caspase-1, IL-1β, and β-actin in BLA, as well as their statistical analysis of relative proteins expression. One-way ANOVA with Tukey’s post hoc test, ***P < 0.001, compared with CONT; ###*P* < 0.001, compared with SUS-PBS; *n* = 3

We also investigated the potential role of pharmacologically inhibiting reverse transcriptases on CUMS-induced microglial morphological activation and biological immuno-inflammation. The morphological results showed that susceptible mice receiving a 2-w Tenofovir, Emtricitabine, Lamivudine, and Rilpivirine significantly decreased the percentage of Iba1^+^CD68^+^ microglia with a larger area of cells and smaller cell bodies in BLA, compared with susceptible mice (Fig. 4A, B). Moreover, susceptible mice receiving a 2-w Tenofovir, Emtricitabine, Lamivudine, and Rilpivirine also strikingly decreased the percentage of CD11b^+^CD45^Low^MHC-II^+^ microglia (Fig. 4C), downregulation of *MuERV-L*, *MusD*, and *IAP* (Fig. 4D), as well as inhibited microglial cGAS–IFI16–STING activation, NF-κB signaling pathway priming, as well as NLRP3 inflammasome activation in BLA, compared with susceptible mice (Fig. 4E). However, no additional impact on microglial p-p53 expression in BLA of those inhibitors- and antagonists-treated mice, compared with susceptible mice (Fig. 4E).

### *p53* knocking-down in BLA reversed chronic stress-induced negative emotional behaviors

**Fig. 5:**
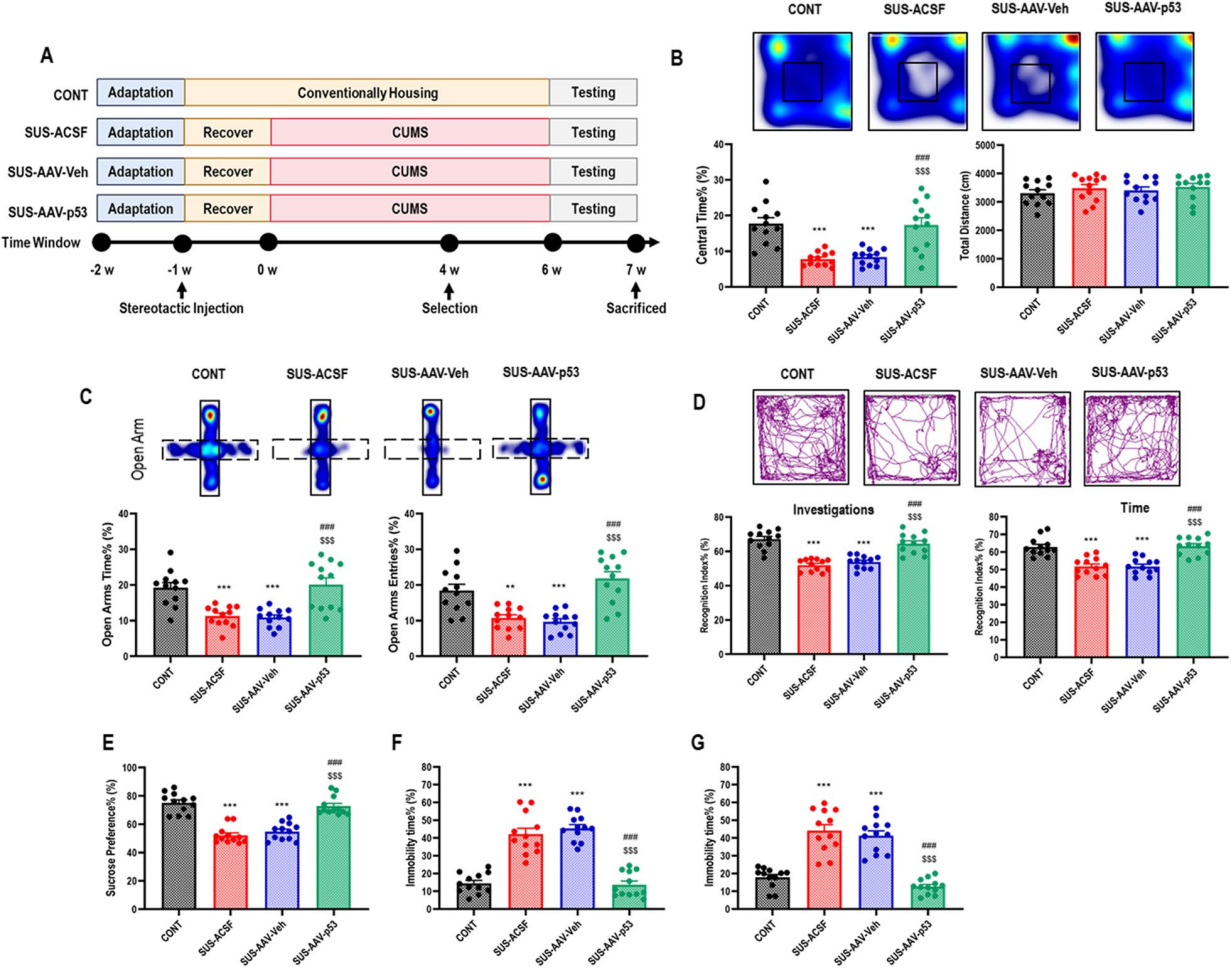
*p53* knocking-down in BLA reversed chronic stress-induced negative emotional behaviors. **A** Schematic representation. **B** Heat maps and statistical analysis of central time% and total distance in OF test for each group. One-way ANOVA with Tukey’s post hoc test, ***P < 0.001, compared with CONT; ###*P* < 0.001, compared with SUS-ACSF; $$$ *P* < 0.001, compared with SUS-AAV-Veh; *n* = 12. **C** Heat maps and statistical analysis of open arms time% and open arms entries% in EPM test for each group. One-way ANOVA with Tukey’s post hoc test, **P < 0.01, ***P < 0.001, compared with CONT; ###*P* < 0.001, compared with SUS-ACSF; $$$ *P* < 0.001, compared with SUS-AAV-Veh; *n* = 12. **D** Track maps and statistical analysis of recognition index of investigations and time in NOR test for each group. One-way ANOVA with Tukey’s post hoc test, ***P < 0.001, compared with CONT; ###*P* < 0.001, compared with SUS-ACSF; $$$ *P* < 0.001, compared with SUS-AAV-Veh; *n* = 12. **E** Statistical analysis of sucrose preference% in SP test for each group. One-way ANOVA with Tukey’s post hoc test, ***P < 0.001, compared with CONT; ###*P* < 0.001, compared with SUS-ACSF; $$$ *P* < 0.001, compared with SUS-AAV-Veh; *n* = 12. **F** Statistical analysis of immobility time% in FS test for each group. One-way ANOVA with Tukey’s post hoc test, ***P < 0.001, compared with CONT; ###*P* < 0.001, compared with SUS-ACSF; $$$*P* < 0.001, compared with SUS-AAV-Veh; *n* = 12. **G** Statistical analysis of immobility time% in TS test for each group. One-way ANOVA with Tukey’s post hoc test, ***P < 0.001, compared with CONT; ###*P* < 0.001, compared with SUS-ACSF; $$$*P* < 0.001, compared with SUS-AAV-Veh; *n* = 12

The evidence that pharmacologic activation of *p53* could occupancy ERVs promoters and inhibit ERVs repressors, as well as antiretroviral therapy and inhibiting ERVs transcription had no role in CUMS-induced *p53* activation, indicated that *p53* activation could accelerate the CUMS-induced microglial immuno-inflammation via promoting ERVs transcription. Therefore, we further investigated the potential role of *p53* knocking-down in BLA on CUMS-induced negative emotional behaviors. The behavioral results showed that mice receiving *p53* knocking-down (Fig. 5A) significantly reversed the negative emotional behaviors indicated by increased center time% in OF test (Fig. 5B), increased open arms time% and open arms entries% in EPM test (Fig. 5C), increased recognition index on investigations and time in NOR test (Fig. 5D), increased sucrose preference% in SP test (Fig. 5E), as well as decreased immobility time% in both FS and TS test (Fig. 5F, G), compared with mice receiving ACSF and AAV-Veh.

### *p53* knocking-down in BLA reversed chronic stress-induced microglial morphological activation, ERVs transcription, intrinsic nucleic acids sensing response, and immuno-inflammation in BLA

**Fig. 6:**
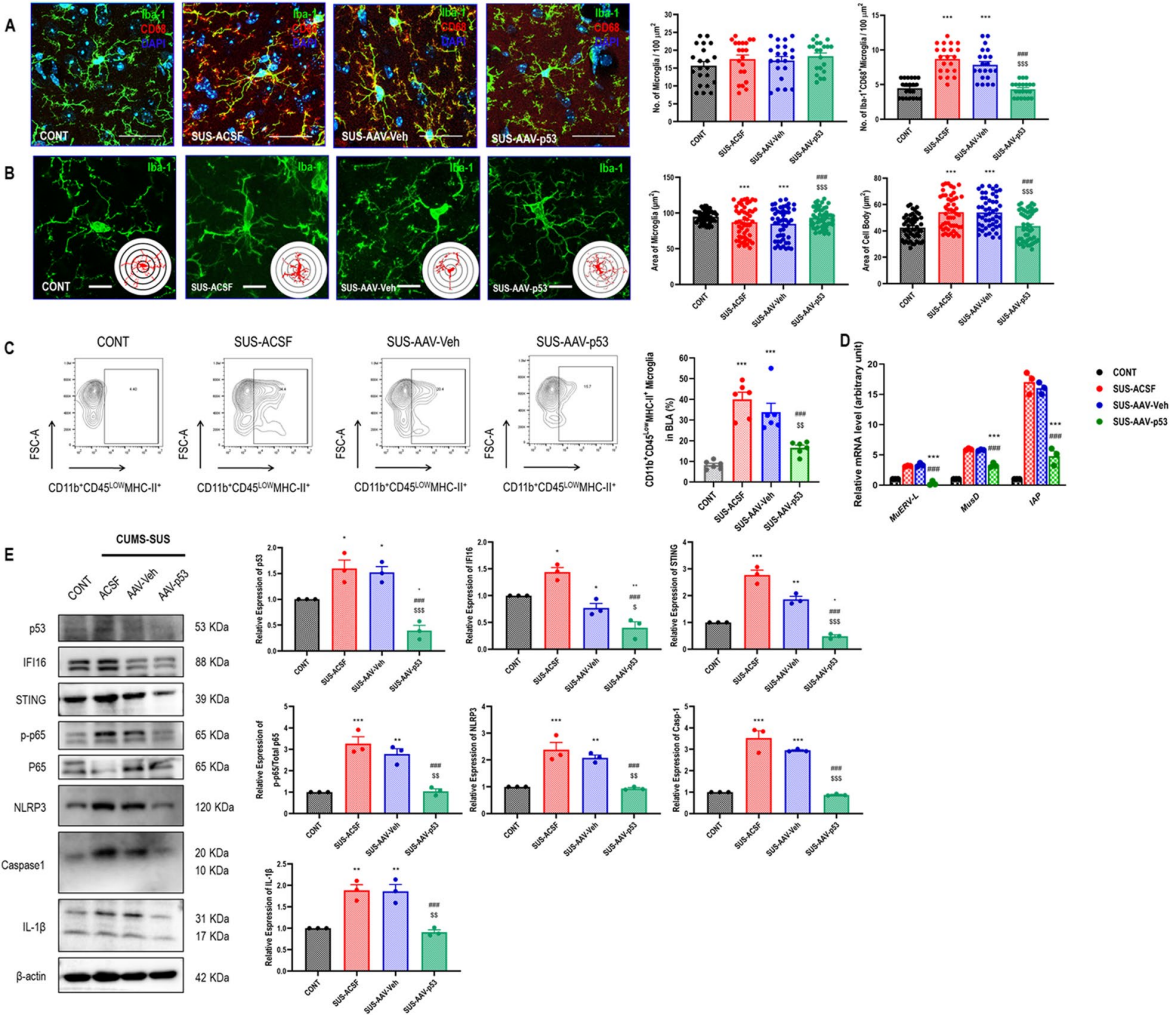
*p53* knocking-down in BLA reversed chronic stress-induced microglial morphological activation, ERVs transcription, intrinsic nucleic acids sensing response, and inflammation in BLA. **A** Representative immunofluorescent images showing Iba1^+^CD68^+^ microglia in BLA, as well as statistical analysis of the number of Iba1^+^ microglia and Iba1^+^CD68^+^ microglia in BLA. One-way ANOVA with Tukey’s post hoc test, ***P < 0.01, compared with CONT; ###*P* < 0.001, compared with SUS-ACSF; $$$ *P* < 0.001, compared with SUS-AAV-Veh; scale bar = 40 × ; a total of 21 replicates from *n* = 3. **B** Representative immunofluorescent and their 3D reconstructive Sholl images showing Iba1^+^ microglia in BLA, as well as statistical analysis of microglial area and cell body area of Iba1^+^ microglia in BLA. One-way ANOVA with Tukey’s post hoc test, ***P < 0.01, compared with CONT; ###*P* < 0.001, compared with SUS-ACSF; $$$*P* < 0.001, compared with SUS-AAV-Veh; scale bar = 60 × ; a total of 90 replicates from *n* = 3. **C** Representative flow cytometry images for showing CD11b^+^CD45^Low^MHC-II^+^ microglia in BLA, as well as statistical analysis of percentage of CD11b^+^CD45^Low^MHC-II^+^ microglia in BLA. One-way ANOVA with Tukey’s post hoc test, ***P < 0.01, compared with CONT; ###*P* < 0.001, compared with SUS-ACSF; $$ *P* < 0.01, compared with SUS-AAV-Veh; *n* = 6. **D** Statistical analysis of murine orthologous microglial ERVs genes expression in BLA. One-way ANOVA with Tukey’s post hoc test, ###*P* < 0.01, compared with SUS-ACSF; $$*P* < 0.01, $$$ *P* < 0.001, compared with SUS-AAV-Veh; *n* = 3. **E** Representative western blot images for showing microglial p-p53, p53, IFI16, STING, p-p65, p65, NLRP3, caspase-1, IL-1β, and β-actin in BLA, as well as their statistical analysis of relative proteins expression. One-way ANOVA with Tukey’s post hoc test, **P < 0.05, **P < 0.01, ***P < 0.001, compared with CONT; ###*P* < 0.001, compared with SUS-ACSF; $*P* < 0.05, $$*P* < 0.01, $$$*P* < 0.001, compared with SUS-AAV-Veh; *n* = 3

Moreover, we also confirmed the protective role of *p53* knocking-down in BLA on CUMS-induced microglial morphological activation and biological immuno-inflammation. The morphological results showed that mice receiving *p53* knocking-down significantly decreased the percentage of Iba1^+^CD68^+^ microglia with a larger area of cells and smaller cell bodies in BLA, compared with mice receiving ACSF and AAV-Veh (Fig. 6A, B). Moreover, mice receiving *p53* knocking-down also strikingly decreased the percentage of CD11b^+^CD45^Low^MHC-II^+^ microglia (Fig. 6C), downregulation of *MuERV-L*, *MusD*, and *IAP* (Fig. 6D), as well as inhibited microglial cGAS–IFI16–STING activation, NF-κB signaling pathway priming, as well as NLRP3 inflammasome activation in BLA, compared with mice receiving ACSF and AAV-Veh (Fig. 6E).

## Discussion

**Fig. 7:**
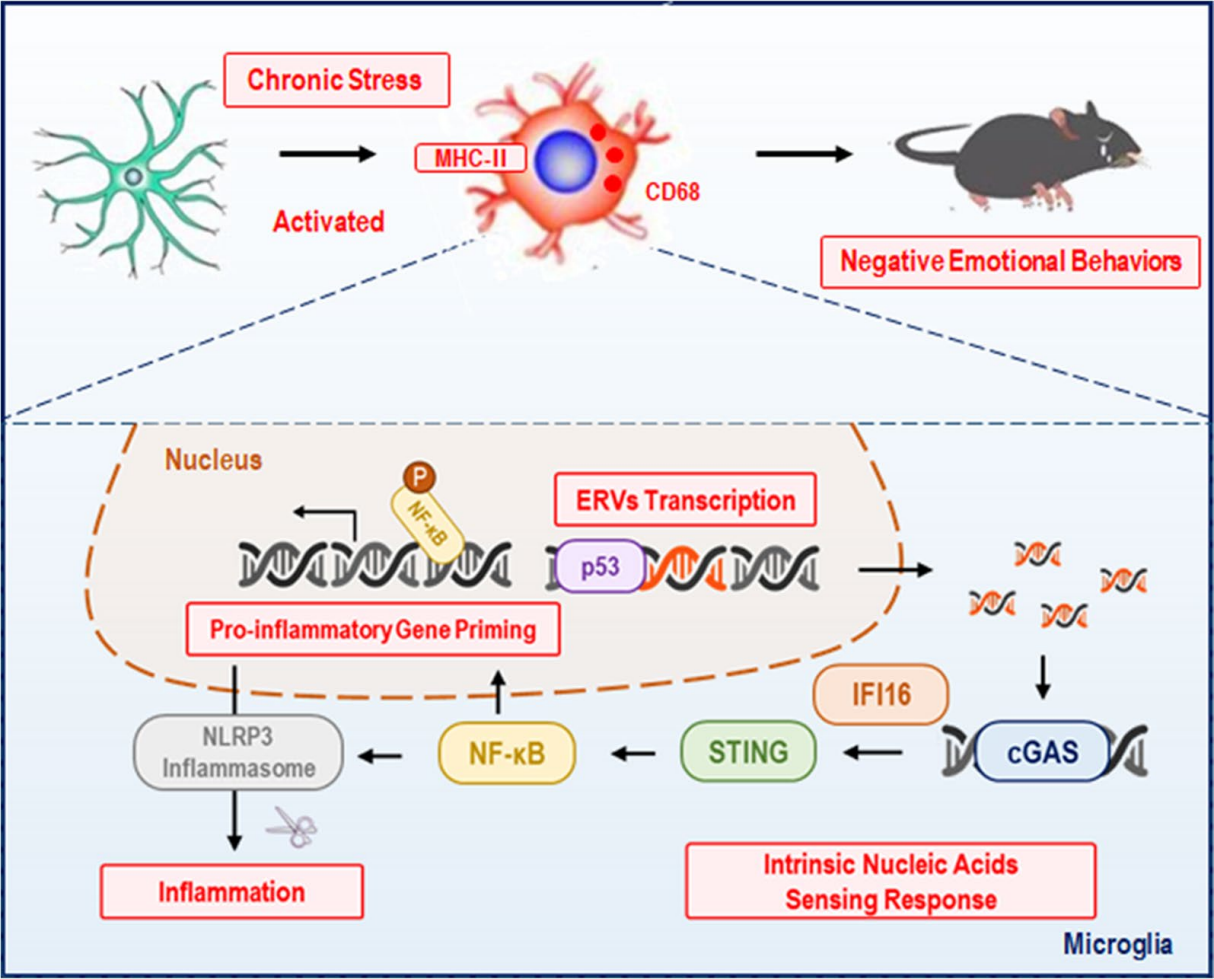
Graphic summary of ERVs-activated microglial immuno-inflammation

Our results suggested that chronic stress induced microglial pro-inflammatory activation, cGAS–IFI16–STING activation, NF-κB signaling pathway priming, as well as NLRP3 inflammasome activation in BLA, and that were associated with negative emotional behaviors, including anxiety and depression. Both antiretroviral therapy and pharmacological inhibition of reverse transcriptases were significantly reversed microglial morphological activation and biological immuno-inflammation, as well as improved negative emotional behaviors. Moreover, knocking-down the ERVs transcriptional regulation gene *p53* also presented a protective role in chronic stress-induced microglial immuno-inflammation and negative emotional behaviors. We provided a graphic summary of the mechanism of ERVs-activated microglial immuno-inflammation in Fig. 7.

A growing body of evidence indicates positive correlation between the increased levels of inflammatory cytokines and psychotic disorders, and that are generally related to various aspects of patients’ behavioral symptomatology, even suicidal ideation and suicide attempts [31]. BLA is the core node connecting midbrain, hippocampus, and cortex within neural network on mediating anxiety and depression. Previous neuroimage studies revealed that the vulnerability of midbrain–amygdala–hippocampus/cortex functional connectivity to local inflammation was contributed to the development of anxiety and depression [32–34]. Microglia, the only resident immune cells in CNS, predominantly contributes to the bilaterally immuno-inflammatory response with neurons during the development of anxiety and depression [35–37]. Evidence from our study and others also revealed that the microglial activation played a critical role in exaggerated immuno-inflammation in CNS and that were contributed to chronic stress-induced negative emotional behaviors [38, 39]. Moreover, we also found that the ERVs-associated microglial immuno-inflammation, in part was via NLRP3 inflammasomes activation-dependent manner. However, the mechanism of triggering and accelerating microglial immuno-inflammation under chronic stress condition are far from being revealed.

Theoretically, integrated ERVs sequences still retain a residual expression capacity and could produce a variety of coding or non-coding RNAs, cytosolic DNAs, and defective proviruses proteins [40]. The LTRs play a crucial role in regulating viral genes' transcription, including promoters, enhancers, and polyadenylation signals during ERVs reverse transcription [40, 41]. However, host genomic guardian (e.g., *p53*) and ERVs repressors (e.g., histone demethylase and DNA methyltransferase) would synergistically repress the repetitive elements’ function [22, 23]. Moreover, RNA-induced silencing complex (RISC) and cytosolic nucleic acids sensors signaling pathways could also be activated and degrade the excessive intracellular nucleic acids to keep the host immune homeostasis in normal condition [42, 43]. Previous studies have demonstrated that more than one third of *p53* binding sites in the human genome have been dispersed by class I human ERVs sequences [44], and pharmacological activation of *p53* induced the activation and transcription of ERVs via epigenetic regulation of lysine specific demethylase 1 (LSD1) and DNA (cytosine-5-)-methyltransferase 1 (DNMT1), together with inhibition of RISC’s function [23]. Our results also confirmed that the murine ERVs genes *MuERV-L*, *MusD*, and *IAP* were significantly upregulated in microglia with chronic stress [22, 45]. Furthermore, all of antiretroviral therapy, pharmacological inhibition of reverse transcriptases, as well as *p53* knocking-down showed a significant inhibition of ERVs genes transcription, and that were associated with the improvement of negative emotional behavior. These results indicated a potential role ERVs activation in triggering and accelerating microglial immuno-inflammation under chronic stress condition, and *p53* presented the positive regulatory role in promoting ERVs transcription.

Recently, cytosolic immunogenic nucleic acids sensing, for instance cGAS–STING pathway, has been increasingly recognized as a primary determinant and potent regulator in neuroimmunological diseases, neurological infections, neurodegenerative diseases, as well as neuro-oncology [46–51]. Endoplasmic reticulum protein STING can be bound and activated by cytosolic nucleic acids sensor cGAS, and then lead to a complex intracellular signaling cascade and nuclear translocation of transcription factors, such as NF-κB, that stimulate the production of various pro-inflammatory molecules, including cytokines, chemokines, type I interferons (IFNs) [52, 53]. Previous studies have observed the activation of microglial cGAS–STING pathway in exogenous viral infections, traumatic brain injury, cerebral ischemic stroke, and Alzheimer's disease [50, 54–57], however, the activators of cGAS–STING pathway were come from exogenous virus or extracellular nucleic acids released by damaged or dead cells that phagocytized by microglia. Our study revealed that the nucleic acids for activating microglial cGAS–STING pathway were derived from excessive endogenous ERVs transcription and their products under chronic stress condition, indicated by the evidence that antiretroviral therapy, pharmacological inhibition of reverse transcriptases, as well as *p53* knocking-down significant inhibited ERVs genes’ expression. Moreover, previous study found that IFI16 also can facilitate STING activation [58], which has been also confirmed in our study.

There are several limitations in our study. First, the specific ERVs products that mediate microglial immuno-inflammation should be identified in the further study. Second, the role of other intracellular ERVs transcriptional products sensors including coding or non-coding RNAs, DNAs, and proteins, such as a verity of endosomal TLRs, should be comprehensively identified in chronic stress-induced microglial immuno-inflammation and negative emotional behaviors. For instance, human ERVs-W Env proteins can stimulate both TLR2 and TLR4 and then induce NF-κB activation and pro-inflammatory cytokine secretion [59, 60]. TLR3 can detect dsRNA from certain human immunodeficiency virus (HIV) strains and even participate their transactivation, replication, and inflammation [61–63]. Moreover, human ERVs ssRNA and HIV guanosine- and uridine-rich ssRNA can be recognized by both TLR7 and TLR8, and then stimulate the production of IFN-α and pro-inflammatory cytokines by immune cells such as dendritic cells (DCs) and macrophages [64–66]. TLR9, the sole DNA-sensing TLR, can specifically recognize unmethylated CpG-rich DNA that is present mostly in bacteria, DNA viruses, and even retrovirus, such as HIV [67–70]. Third, given that mouse genomes, in particular, have many active ERVs, which contrasts strikingly with the human genome [45], the role of human ERVs-associated microglial immuno-inflammation should be further identified in chronic stress-induced negative emotional behaviors. Last, although previous study has suggested that a human ERV genes and their products could alter glutamate synapse maturation and generate behavioral deficits [71], further supporting the possible etiological interplay between genetic, immune, and synaptic factors in psychosis, the concern should be highlighted that the present study is based on a rodent model and that major differences with human ERVs and with genes controlling their repression or expression in humans are far from being revealed. Therefore, the detailed pathways and effectors from ERVs to neuronal dysfunctions via microglia and neuroinflammation, should be left open for a translation onto humans.

Together, our study concluded that chronic stress induced negative emotional behaviors were associated with microglial immuno-inflammation mediated by ERVs-activated cGAS–IFI16–STING–NF-κB–NLRP3 in BLA. Antiretroviral therapy, pharmacological inhibition of reverse transcriptases, as well as knocking-down the ERVs transcriptional regulation gene *p53* presented a protective role in chronic stress-induced microglial immuno-inflammation and negative emotional behaviors. These results provided an innovative therapeutic approach that targeting ERVs-associated microglial immuno-inflammation may be potentially beneficial to the patients with psychotic disorders.

## Supplementary Information

**Additional file 1: Table S1**. The safety of antiretroviral therapy (*n*=3).

## Data Availability

The authors declare that all data supporting the findings of this study are available within the article and from the corresponding author upon reasonable request.
