# Supplementary material for: Activation of endogenous retrovirus triggers microglial immuno-inflammation and contributes to negative emotional behaviors in mice with chronic stress
Source: J Neuroinflammation. 2023 Feb 15;20:37. doi: 10.1186/s12974-023-02724-x (PMC9933381; doi:10.1186/s12974-023-02724-x)
Supplement: Supplementary file 1 — Additional file 1: Table S1. The safety of antiretroviral therapy (n=3). [file 12974_2023_2724_MOESM1_ESM.docx]

**Table S1** The Safety of antiretroviral therapy (n=3).

| **Items** | **CONT** | **SUS-PBS** | **SUS-ET** | **Units** | **Reference** |
| --- | --- | --- | --- | --- | --- |
| WBC | 5.4±0.8 | 7.6±1.3 | 2.4±1.2 | 10^9/L | 0.8-6.8 |
| Lymph# | 3.0±0.6 | 4.9±0.8 | 1.4±0.5 | 10^9/L | 0.7-5.7 |
| Mon# | 0.2±0.0 | 0.3±0.0 | 0.1±0.0 | 10^9/L | 0.0-0.3 |
| Gran# | 2.2±0.1 | 2.4±0.1 | 0.9±0.0 | 10^9/L | 0.1-1.8 |
| Lymph% | 55.8±2.7 | 64.2±4.1 | 59.0±3.3 | % | 55.8-90.6 |
| Mon% | 3.7±0.7 | 4.6±0.4 | 3.8±0.4 | % | 1.8-6.0 |
| Gran% | 40.5±4.1 | 31.2±5.3 | 37.2±3.9 | % | 8.6-38.9 |
| RBC | 9.21±1.0 | 9.22±0.6 | 9.59±0.9 | 10^12/L | 6.36-9.42 |
| HGB | 153±4.7 | 151±5.2 | 155±3.9 | g/L | 110-143 |
| HCT | 49.1±2.4 | 49.8±2.0 | 51.2±1.3 | % | 34.6-44.6 |
| MCV | 53.4±3.3 | 54.1±3.5 | 53.4±4.0 | fL | 48.2-58.3 |
| MCH | 16.6±0.7 | 16.3±1.2 | 16.1±1.1 | pg | 15.8-19 |
| MCHC | 311±10.3 | 303±13.6 | 302±12.9 | g/L | 302-353 |
| RDW | 14.6±0.6 | 14.0±0.2 | 14.3±0.3 | % | 13-17 |
| PLT | 1365±27.3 | 1666±33.3 | 1662±29.7 | 10^9/L | 450-1590 |
| MPV | 5.8±0.1 | 6.7±0.2 | 6.2±0.2 | fL | 3.8-6.0 |
| PDW | 16.1±0.5 | 16.5±0.7 | 16.3±0.5 | —— | —— |
| PCT | .*** | .*** | .*** | % | —— |
